# Anomaly Detection Based on Time Series Data of Hydraulic Accumulator

**DOI:** 10.3390/s22239428

**Published:** 2022-12-02

**Authors:** Min-Ho Park, Sabyasachi Chakraborty, Quang Dao Vuong, Dong-Hyeon Noh, Ji-Woong Lee, Jae-Ung Lee, Jae-Hyuk Choi, Won-Ju Lee

**Affiliations:** 1 Division of Marine Engineering, Korea Maritime and Ocean University, Busan 49112, Republic of Korea; 2 Interdisciplinary Major of Maritime and AI Convergence, Korea Maritime and Ocean University, Busan 49112, Republic of Korea; 3 Terenz Co., Ltd., Busan 48060, Republic of Korea; 4 Division of Marine System Engineering, Korea Maritime and Ocean University, Busan 49112, Republic of Korea; 5 Hwajin Enterprise Co., Ltd., 25, Mieumsandan 2-ro, Gangseo-gu, Busan 46748, Republic of Korea

**Keywords:** accumulator, pulsating pressure data, CNN, autoencoder, anomaly detection

## Abstract

Although hydraulic accumulators play a vital role in the hydraulic system, they face the challenges of being broken by continuous abnormal pulsating pressure which occurs due to the malfunction of hydraulic systems. Hence, this study develops anomaly detection algorithms to detect abnormalities of pulsating pressure for hydraulic accumulators. A digital pressure sensor was installed in a hydraulic accumulator to acquire the pulsating pressure data. Six anomaly detection algorithms were developed based on the acquired data. A threshold averaging algorithm over a period based on the averaged maximum/minimum thresholds detected anomalies 2.5 h before the hydraulic accumulator failure. In the support vector machine (SVM) and XGBoost model that distinguish normal and abnormal pulsating pressure data, the SVM model had an accuracy of 0.8571 on the test set and the XGBoost model had an accuracy of 0.8857. In a convolutional neural network (CNN) and CNN autoencoder model trained with normal and abnormal pulsating pressure images, the CNN model had an accuracy of 0.9714, and the CNN autoencoder model correctly detected the 8 abnormal images out of 11 abnormal images. The long short-term memory (LSTM) autoencoder model detected 36 abnormal data points in the test set.

## 1. Introduction

A hydraulic accumulator is a critical component that maintains constant pressure, stores and recaptures energy, powers chassis suspensions, reduces pressure peaks, and dampens shock, vibration, and pulsations to make the mechanical system run smoothly.

In this critical role, hydraulic accumulators are used in a wide range of specific applications, such as in construction fields and in hydro-pneumatic boom suspension systems, where accumulators are used for telehandlers. In oil and gas fields, accumulators provide instantaneous hydraulic power for emergency safety functions of blowout preventers used on drilling rigs and valve actuators in well control mechanisms. In the energy field, accumulators are used in hydraulic pitch or brake systems for wind turbines and hydraulic pitch rotator systems for solar energy. In the agricultural field, the energy storage capability of an accumulator provides road-friendly suspension and pulsation dampening. In automotive applications, accumulators are used in transmission systems. In the maritime field, the accumulator is mounted on an engine hydraulic cylinder unit that operates an engine fuel injection and exhaust system. This alleviates the high-pressure shock and pulsation during engine operation, keeps the engine system pressure constant, and dampens vibrations to ensure smooth engine operation [1].

However, despite such uses, hydraulic accumulators have raised the following problems. Lindák et al. mentioned that all systems containing liquid-flowing pipes might experience pressure pulses with a change in the flow rate. This phenomenon is caused because the changes in the kinetic energy of liquids are converted into pressure changes. These are then spread in the form of waves in the liquids and may cause irreparable damage to the installation [2]. Some accumulators suffer from breakage, and parts of the accumulator body are ejected into the engine room, causing damage to the engine. Accordingly, such breakage poses a severe threat to property and people [3].

Owing to the persistent problems of the accumulator, one of the leading diesel engine manufactures, MAN Energy Solutions, has been updating the following recommendations: (1) Installing protective guards on accumulators; (2) appropriate tightening of the screws fastening the accumulator; (3) regular checks of the nitrogen pressure; (4) replacing the accumulator if it has been in operation without nitrogen pressure [3,4,5]. However, these measures do not entirely prevent the breakage of the accumulator. Therefore, this study conducted a preliminary investigation into applying artificial intelligence to a hydraulic system to develop an algorithm to detect the breakage of the accumulator in advance.

El-Betar et al. employed a feedforward neural network to diagnose two common faults in a hydraulic power system, namely actuator internal leakage and valve spool blockage [6]. Pichler et al. tested the naive, feature engineering, image transformation, and statistical feature extraction approaches for detecting faults in a hydraulic accumulator loading circuit [7]. Gareev et al. compared the accuracy of four machine-learning approaches for fault detection in a hydraulic system. The first three approaches are based on support vector machine (SVM) classifiers with linear, polynomial, and radial basis function kernels, and the last is gradient boosting on oblivious decision trees [8]. Kim and Jeong used a five-layer VGGNet based on a convolutional neural network (CNN) model for data augmentation because the amount of hydraulic system data is insufficient, which shows good performance in terms of accuracy and loss for the classification of hydraulic system data [9]. Huang et al. proposed a CNN-based fault diagnosis method for hydraulic systems by designing a deep learning model with a multirate data sample and visualized the analysis with t-distributed stochastic neighbor embedding [10]. Rosaperez proposed a self-learning methodology that uses a deep learning model to predict failures in a hydraulic accumulator, and it was possible to forecast its breakdown two weeks in advance [11].

This study was conducted to prevent equipment failure, personal injury, and ship operation loss by detecting in advance the situation in which the marine hydraulic accumulator is broken by extreme pulsating pressure. After referring to the studies on the development of algorithms for anomaly detection above, in this study, hydraulic pulsating pressure data were acquired from a hydraulic accumulator used in a real ship, and six algorithms were developed to detect the anomalies based on the acquired data.

## 2. Materials and Methods

### 2.1. Description of Marine Hydraulic Accumulators and Their Challenges

Marine hydraulic accumulators are mounted on engine hydraulic cylinder units that operate the engine fuel injection and exhaust systems. This is a critical part of a marine engine that maintains a constant engine system pressure by alleviating the high-pressure shock and pulsation during engine operation, enabling smooth engine operation by dampening vibrations.

**Figure 1:**
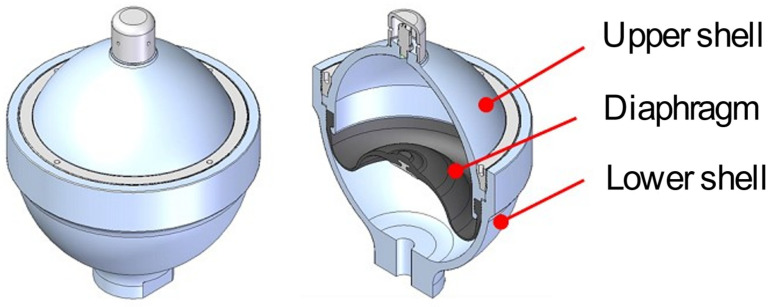
Structure of the marine hydraulic accumulator.

As shown in Figure 1, the marine hydraulic accumulator consists of hemispherical upper and lower shells, and the fluid and gas are separated by a diaphragm made of synthetic rubber. The nitrogen recommended by the manufacturer is filled under an appropriate amount of pressure between the upper shell and diaphragm, and the lube oil of the hydraulic system is filled between the diaphragm and lower shell. The capped part of the uppermost part of the upper shell is a port for filling nitrogen.

When there is a problem with the control unit of an electronically controlled engine, the marine hydraulic accumulator compensates for the appropriate flow rate and pressure to prevent damage to the components of the hydraulic cylinder unit and helps in smooth machine operation.

The marine hydraulic accumulator is usually operated under extreme pressure pulsation conditions. If the pressure pulsation within the engine is abnormally severe and continuous abnormal pressure pulsation occurs owing to the mechanical failure of the engine or abnormal control system, the stress amplitude of the hydraulic accumulator becomes very large. This reduces fatigue life rapidly and causes damage. When the marine hydraulic accumulator is damaged, the normal function of the engine becomes impossible; accordingly, the engine must be stopped, and the hydraulic accumulator should be maintained or replaced. Therefore, in order to increase the safety of a ship, it is necessary to develop an algorithm for the hydraulic accumulator that can detect abnormalities before breakage.

### 2.2. Experimental Setup

**Figure 2:**
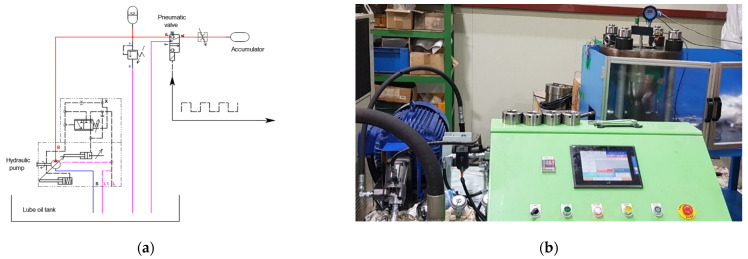
(**a**) Schematic diagram of the hydraulic system for the hydraulic accumulator test; (**b**) experimental setup for testing the hydraulic accumulator.

Figure 2a shows the schematic of the hydraulic accumulator experiment. The blue line indicates that the lube oil in the lube oil tank travels to the hydraulic pump. The red line indicates the lube oil pressurized by the hydraulic pump going to the accumulator. If the pressure of the lube oil in the red line exceeds a certain range, it is bypassed to the lube oil tank, as indicated by the pink line. The lube oil in the red line then arrives at the pneumatic valve. According to the pulse signal cycle set by the user, the pressurized lube oil enters the lower shell of the accumulator and is bypassed to the lube oil tank continuously until the pulse signal is stopped or the accumulator is broken.

Figure 2b shows the experimental setup of the hydraulic system, and the hydraulic accumulator is shown in Figure 2a. The experiment was conducted with a hydraulic accumulator in which only the lower and upper shells were assembled without a diaphragm so that the inside was filled with only the working fluid. On the green control panel, the opening and closing of the pneumatic valve were set to 0.4 s. The hydraulic pressure supplied by the hydraulic pump was set to 400 bar, the upper limit was set to 450 bar, and the lower limit was set to 40 bar to ensure the safety of the experimental equipment. In this case, when the accumulator breaks, the hydraulic pressure is rapidly reduced to less than 40 bar; thus, it is possible to prevent the lube oil from being sprayed by shutting down the experimental equipment. Since 400 bar of high-pressure lube oil is supplied from the lower part of the accumulator, the cover for the hydraulic accumulator is firmly fastened with studs and nuts for safety.

A digital pressure sensor was installed in the nitrogen-filling port of the accumulator to collect the pressure pulsation data. The detailed specifications of the digital pressure sensors are listed in Table 1.

### 2.3. Theory of Algorithm Used for Anomaly Detection

#### 2.3.1. SVM

The SVM is a model that defines and calculates a decision boundary for data classification. When the data are of a higher dimension, the decision boundary becomes a hyperplane rather than a line. Kernel tricks are used to convert low-dimensional data into high-dimensional data, and an RBF kernel trick, such as Equation (1), was used in this study. Here, $x_{i},\;x_{j}$ are the input vectors, and *γ* is a hyperparameter that plays the role of regulation. Equation (1) is as follows:

(1) $$K\left(x_{i},\;x_{j}\right)=\exp\left(-\gamma ∥x_{i}-x_{j}∥{}^{2}\right),\;\;\gamma >0$$

A hyperplane that performs the optimal classification of high-dimensional data is obtained using Equation (2). In the lower part of Equation (2), $x_{i}$ (i=1, 2, …, M) is the input data, w is an M-dimensional vector, M is the number of samples, and b is scalar. The vectors w and b were used to define the position of the separating hyperplane. The samples were assumed to have two classes, namely positive and negative. Each class associated with the labels either is $y_{i}=1$ for the positive class or $y_{i}=-1$ for the negative class. In the upper part of Equation (2), C is the error penalty, and $\xi_{i}$ is a slack variable that measures the distance between the margin and the example $x_{i}$ [12]. Equation (2) is as follows:

(2) $$\begin{array}{c} \mathrm{minimize}\;\frac{1}{2}\;{∥w∥}^{2}+C\sum_{i=1}^{M}\xi_{i},\\ \mathrm{subject}\;\mathrm{to}\;\left\{\begin{array}{c} y_{i}\left(w^{T}x_{i}+b\right)\;\geq 1-\xi_{i}\;,\;\;\;\;\;i=1,\;2,\;\ldots ,\;M\;\\ \;\;\;\xi_{i}\geq \;0,\;\;\;\;\;\;\;\;\;\;\;\;\;\;\;\;\;\;\;\;\;\;\;\;\;\;\;\;\;\;\;\;\;i=1,\;2,\;\ldots ,\;M\;\;\; \end{array}\right. \end{array}$$

The SVM defines a decision boundary using support vectors, which are the points of each class closest to the decision boundary, and it classifies the unclassified points by comparing them with the corresponding decision boundary. The distance between the support vector and the decision boundary is called the margin, and the SVM attempts to make the maximum possible margin within the allowable error range.

#### 2.3.2. XGBoost

The XGBoost [13] model uses base learner as a decision tree, and the training proceeds in a manner that uses the residuals to supplement the weaknesses of the previous model. Although XGBoost is based on gradient boosting, in terms of performance, it is superior to existing gradient boosting models. The original gradient boosting model built trees as a series, whereas XGBoost builds trees in parallel [14]. New models were recursively added to adjust for errors made by existing models until no further performance improvement was observed. The newly created models predict the residuals of the prior models and are added to obtain the final prediction. In XGBoost, the gradient descent algorithm minimizes loss when adding new models. Furthermore, XGBoost has various hyperparameters, which are tuned to further improve the performance [15].

#### 2.3.3. CNN

**Figure 3:**
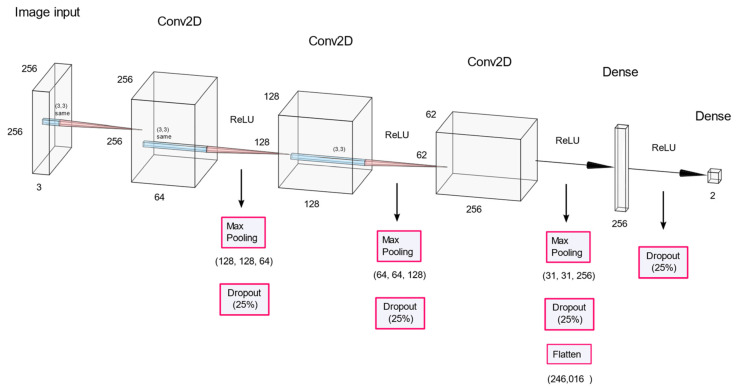
Representation of the CNN structure used in this study, in the AlexNet format.

The CNNs were first used in the study of the visual cortex of the brain and have been used in image recognition since the 1980s. Figure 3 shows the CNN model used in this study to enhance the schematic understanding of the CNN. The input image is a pulsating pressure graph picture, which has a size of 256 × 256 pixels and three channels of red, green, and blue (RGB).

The CNN is a series of convolution operations, and the output calculation of neurons in the convolution layer is the same as in Equation (3). Here, $z_{i,j,k}$ is the output of the neuron located at row i and column j in the k feature map of Conv2D, which is a convolutional layer. Furthermore, $s_{h}$ and $s_{w}$ are the vertical and horizontal strides, respectively; $f_{h}$ and $f_{w}$ are the height and width of the kernel, respectively. A matrix in which a specific number for the convolution operation is combined is called a kernel or filter, and the interval in which the kernel is applied is called a stride. Here, $f_{n^{\prime }}$ is the number of feature maps in the previous convolutional layer, while $x_{i^{\prime },j^{\prime },\;k^{\prime }}$ are the outputs of the neurons in row $i^{\prime }$, column $j^{\prime }$, and $k^{\prime }$ feature maps of the previous convolutional layer, respectively. Additionally, $b_{k}$ is the bias of the k feature map in the current convolutional layer, while $w_{u,\;v,k^{\prime },k}$ is the connection weight between all neurons in the k feature map of the current convolutional layer and the inputs located in the u row, v column, and $k^{\prime }$ feature map associated with the neuron kernel [16]. Equation (3) is as follows:

(3) $$z_{i,j,k}=b_{k}+\overset{f_{h}-1}{\underset{u=0}{\sum }}\overset{f_{w}-1}{\underset{v=0}{\sum }}\overset{f_{n^{\prime }}-1}{\underset{k^{\prime }=0}{\sum }}x_{i^{\prime },j^{\prime },k^{\prime }}\times w_{u,v,k^{\prime },k}\;\;\;\;\;\;\;,\;\;\;\;\;\;\;where\left\{\begin{array}{c} i^{\prime }=i\times s_{h}+u\\ j^{\prime }=j\times s_{w}+v \end{array}\right.\;$$

Padding is performed before the convolution operation, which adds rows and columns as the specified number of widths to the edge of the input. Zero padding is mainly used to fill the value with zero and, in this study, zero padding was performed to maintain the same height and width even after convolution.

Subsequently, the result of the convolution operation is passed through the activation function ReLU in Equation (4). If x is greater than zero, the slope is one; if it is less than zero, the function value is zero. Equation (4) is as follows:

(4) $$f\left(x\right)=\max\left(0,\;x\right)$$

Pooling is the creation of a subsample of the input image to reduce the number of parameters. Pooling, as with convolution, requires specifying the size, stride, and padding type; however, pooling has no weights. Instead, pooling uses summation functions, such as max or average.

Dropout is a technique that removes neurons with a probability between zero and one, and it is used to prevent overfitting that can occur by over-focusing on only a specific feature.

The number of final output neurons of the CNN structure that distinguishes images is usually two or more. When two images are to be distinguished, the sigmoid function of Equation (5), which outputs a value between zero and one, is used. Equation (5) is as follows:

(5) $$sigmoid\left(x\right)=\frac{1}{1+e^{-x}}$$

#### 2.3.4. Autoencoder

An autoencoder automatically extracts the nonlinear features or output similar to the input. The autoencoder consists of a neural network, and the basic autoencoder consists of input, hidden, and output layers. The number of neurons in each layer is n, m, and n, respectively, and m must be less than n. The part composed of the input and hidden layers is called the encoder, and the part composed of the hidden and output layers is called the decoder. The encoder compresses high-dimensional input data $x=\left\{x_{1},\;x_{2},\;\ldots ,\;x_{n}\right\}$ into low-dimensional $h=\left\{h_{1},\;h_{2},\;\ldots ,\;h_{m}\right\}$ by function f, as shown in Equation (6). Here, $s_{f}$ is the activation function, W is the weight matrix of size m × n, and $b\in R^{m}$ is the bias vector. Equation (6) is as follows:

(6) $$h=f\left(x\right)=s_{f}\left(Wx+b\right)$$

The decoder reconstructs the hidden representation h through function g into $x^{\prime }=\left\{x_{1}^{\prime },\;x_{2}^{\prime },\;\ldots \;,\;x_{n}^{\prime }\right\}$ of the same size as the input data, as shown in Equation (7), where $s_{g}$ is the activation function of the decoder, $W^{\prime }$ is the weight matrix of size n × m, and $b^{\prime }\in R^{n}$ is a bias vector. Equation (7) is as follows:

(7) $$x^{\prime }=g\left(h\right)=s_{g}\left(W^{\prime }h+b^{\prime }\right)$$

The autoencoder is trained to minimize the reconstruction error between x and $x^{\prime }$ [17].

#### 2.3.5. LSTM Autoencoder

The LSTM autoencoder uses the LSTM structure, and the LSTM cell can be mathematically expressed as Equation (8). The first line of Equation (8) represents the forget gate in the LSTM cell. When $f_{t}$ is one, the information is maintained, and when $f_{t}$ is zero, the information is removed. In Equation (8), $x_{t}$ and $h_{t}$ denote the input and recurrent information, respectively; $W_{h}$ and $W_{x}$ are the weights; b is the bias; and σ is a sigmoid function. The sigmoid layer, called the input gate layer, in the second line of Equation (8), determines which value to update. Subsequently, the tanh layer of the third line of Equation (8) creates a vector called ${\tilde{c}}_{t}$, which becomes a new candidate value. The fourth line of Equation (8) indicates the state of the LSTM cell. The input data enters the sigmoid layer of the fifth line in Equation (8), and the output information is determined. The cell state is placed in the tanh layer, multiplied by the output of the sigmoid layer, and exported as an output, as shown in the sixth line of the following Equation (8) [18]:

(8) $$\begin{array}{c} f_{t}=\sigma \left(W_{fh}h_{t-1}+W_{fx}x_{t}+b_{f}\right),\\ i_{t}=\sigma \left(W_{ih}h_{t-1}+W_{ix}x_{t}+b_{i}\right),\\ {\tilde{c}}_{t}=tanh\left(W_{\tilde{c}h}h_{t-1}+W_{\tilde{c}x}x_{t}+b_{\tilde{c}}\right),\\ c_{t}=f_{t}\cdot c_{t-1}+i_{t}\cdot {\tilde{c}}_{t},\\ o_{t}=\sigma \left(W_{oh}h_{t-1}+W_{ox}x_{t}+b_{o}\right),\\ h_{t}=o_{t}\cdot \tanh(c_{t}). \end{array}$$

The LSTM autoencoder is composed of an LSTM network for the encoder and decoder and has a symmetrical structure based on the intermediate layer. The LSTM is an algorithm suitable for time-series prediction or anomaly detection because it learns long sequence data well. Therefore, when time-series data within the abnormal range are input to the LSTM autoencoder model trained with time-series data within the normal range, an error occurs between the input and output data; thus, anomaly detection is possible [19].

#### 2.3.6. Performance-Evaluation Metric

To evaluate the performance of the model using the training and test sets, the accuracy of Equation (9) was used as the performance evaluation metric. A description of each element in Equation (9) is listed in Table 2. Equation (9) is as follows:

(9) $$\mathrm{accuracy}=\frac{\mathrm{TP}+\mathrm{TN}}{\mathrm{TP}+\mathrm{FP}+\mathrm{FN}+\mathrm{TN}}$$

## 3. Modeling and Result

### 3.1. Data Analysis

**Figure 4:**
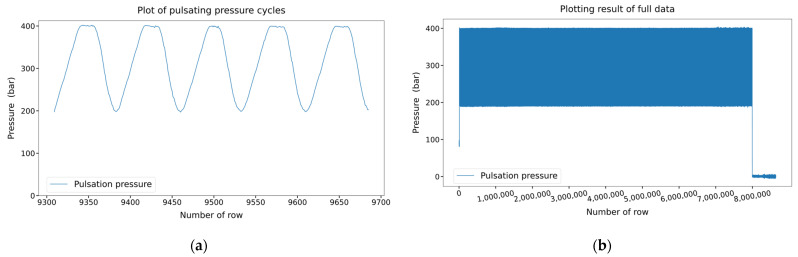
Visualization of pulsating pressure. (**a**) Visualization of five pulsating pressure cycles; (**b**) visualization of the complete data of the pulsating pressure.

The collected pulsating pressure data had 8,640,140 rows and 2 columns. The first column indicates the time, and the second column indicates the pulsating pressure. We obtained 100 rows of pulsating pressure data per second, and the results of plotting the pulsating pressure cycles are shown in Figure 4a. As mentioned in Section 2.2, the on/off state of the pneumatic valve is operated for 0.4 s each, making one cycle 0.8 s long. Since 100 data samples are received in 1 s, 80 data samples are received in 0.8 s. Referring to Figure 4a, 80 rows form 1 cycle, and it can be observed that the data were received well according to the operation setting of the pneumatic valve.

The plotting results of the pulsating pressure cycles in Figure 4a over the entire dataset are shown in Figure 4b. Figure 4b shows that the pulsating pressure was maintained between 200 and 400 bar and then dropped to zero near the 8,000,000th row. In the 7,995,460th row, the accumulator broke, and pressure was not generated, dropping the pressure to zero. The upper and lower limits of the pulsating pressure tend to increase slightly after the 7,000,000th row. This change occurred approximately 2.7 h before the breakage of the accumulator. If an algorithm that can capture this trend is developed, an accident can be prevented using an anomaly detection algorithm. Therefore, in this study, an algorithm was developed to detect such anomalies in advance.

### 3.2. Threshold Averaging Algorithm over a Period

**Figure 5:**
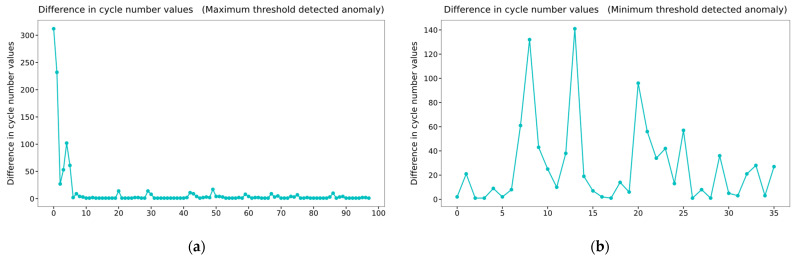
Plotting results of differences in cycle number values. (**a**) Values related to the maximum threshold; (**b**) values related to the minimum threshold.

Up to the 4,000,000th row in Figure 4b corresponds to half of the data before the breakage of the accumulator. Therefore, in this section, the maximum and minimum thresholds from the data up to the 4,000,000th row will be determined, and anomaly detection for the rest of the data will be performed. We placed the maximum and minimum thresholds in the first 50 pulsation cycles into two arrays of the NumPy library to find the thresholds. The previous process was performed in the subsequent 50 cycles, and this process was repeated until the data of the 4,000,000th row. The values in the two arrays were then averaged to obtain the maximum and minimum thresholds. The average thresholds were obtained as 400.4579 and 189.7104, respectively. When the thresholds of the cycles after the 4,000,000th row deviate from 0.2% of the averaged threshold, the number of cycle values is inserted into 2 new arrays. In other words, cycles exceeding 1.002 times the maximum average threshold or less than 0.998 times the minimum average threshold are detected. Subsequently, the difference between the values in each array was calculated, and their plotting results are shown in Figure 5. The first point in Figure 5a indicates that there are more than 300 normal cycles between the cycles in which the abnormality in the maximum threshold is detected. Since these anomalies can occur occasionally, it is not appropriate to use these cycles for anomaly detection. However, from the 10th point, it can be observed that there is almost no difference between the detected cycle numbers, which means that abnormality detection occurs frequently. Therefore, the user can predict that a problem will occur in the accumulator, as there is no difference in values from the 10th to the 20th points based on the plotting result. If it is assumed that the user detects an abnormality at the 20th point, the user predicts a failure 2.5 h before the breakage. This is the case in Figure 5b, which shows the difference between the values of the minimum threshold cycle number detected as an anomaly. The values are not constant, so abnormal detection cannot be performed.

### 3.3. Anomaly Detection Using SVM

Considering the change in pulsation pressure after the 7,000,000th row in Figure 4b, an abnormality was detected by classifying before and after the 7,000,000th row as normal or abnormal. The distinction between normal and abnormal based on the 7,000,000th row was equally applied to Section 3.3, Section 3.4, and Section 3.5. When the front part of the graph in Figure 4b was enlarged, the minimum threshold showed a tendency to gradually decrease until the 25,000th row; therefore, data from the 25,000 to 6,955,000 rows were used.

Among the data from the 25,000th row to the 6,955,000th row, the pulsating pressure data for 15 min were successively contained in a NumPy array. Consequently, a NumPy array with 77 rows and 90,000 columns containing normal pulsating pressure data was created. The data from the 6,955,000th row to the 7,945,000th row were successively contained in a new NumPy array at 15 min intervals, and a NumPy array containing 11 rows and 90,000 columns of abnormal pulsating pressure data was generated. Training and test sets were created at a rate of 60% and 40%, respectively, from the NumPy array of normal pulsating pressure data, and another training set and a test set were created at the same rates from the NumPy array of abnormal pulsating pressure data. These training and test sets were then combined to create a final training set and a test set with normal and abnormal pulsating pressure data. Finally, a training set with 53 rows and 90,000 columns and a test set with 35 rows and 90,000 columns were generated.

Referring to Figure 4b, it can be seen that the maximum threshold value spikes in the section with abnormal pulsating pressure. Therefore, 100 pulsating pressure data were extracted from 90,000 pulsating pressure data for each row in the training and test sets by sorting them in the order of the largest value. Consequently, a training set with 53 rows and 100 columns and a test set with 35 rows and 100 columns were generated. By doing this, the machine learning model was trained with the maximum threshold values of the normal pulsating pressure data and the spike values of the maximum threshold for the abnormal pulsating pressure data. The training and test sets were labeled zero and one for normal and abnormal, respectively.

The generated training and test sets were subjected to standard scaling, as shown in Equation (10) for standardization. In Equation (10), x is the sample, μ is the mean of the samples, σ is the standard deviation of the samples, and z is a standardized sample. Equation (10) is as follows:

(10) $$z=\frac{x-\mu }{\sigma }$$

The scaled training set and training set labels were used as the training data for the support vector classification (SVC) model of scikit-learn library. Among the hyperparameters of the SVC model, C, a regularization parameter, was set to one, and the RBF was used for the kernel trick.

As a result of the performance evaluation of the trained SVC model using the scaled training set and labels of the training set, the accuracy was 0.9811. The performance of the model was evaluated with the scaled test set and the label of the test set, and the accuracy was 0.8571, indicating that the SVC model properly detected anomalies.

### 3.4. Anomaly Detection Using XGBoost

The same training and test sets obtained in Section 3.3 were used for the XGBoost model. The XGBClassifier of the DLMC XGBoost library was used to solve the classification problem. The hyperparameters used in the XGBClassifier model and their values are listed in Table 3. The description of the hyperparameters in Table 3 is as follows. Variable eta represents the learning rate, and the lower the value of eta, the more robust the model and the better the prevention of overfitting. If eta is large, then the update rate of the model increases. Here, min_child_weight is the minimum sum of the instance weights required in a child. Furthermore, gamma is the minimum loss reduction value that determines the further division of a leaf node in the tree. The maximum depth of the tree was set as max_depth, while colsample_bytree is the subsample ratio of columns when constructing each tree. The lambda is an L2 regularization term for the weights, while alpha is the L1 regularization term of the weights. Finally, scale_pos_weight controls the balance between the positive and negative weights [20].

The XGBClassifier model with the above-mentioned hyperparameters was trained using the scaled training set and labels of the training set. As a result of measuring the performance of the XGBClassifier model trained with the scaled training set and the labels of the training set, the accuracy was one. The performance was measured with the scaled test set, and the accuracy was 0.8857 for the label of the test set. Referring to accuracy, which is a performance evaluation metric, the XGBClassifier model is better at detecting anomalies than the SVC model.

### 3.5. Anomaly Detection Using CNN

Considering the change in pulsating pressure after the 7,000,000th row, an abnormality was detected by classifying before and after the 7,000,000th row as normal/abnormal. When the front part of the graph in Figure 4b was enlarged, the minimum threshold was not constant before the 25,000th row; therefore, data from the 25,000th row to the 6,955,000th row were used.

From the 25,000th to the 6,955,000th row, sequential 15 min pulsating pressure data graphs were saved as jpg files with a size of 256 × 256 pixels by using Matplotlib library. The saved files were normal graphs, and 77 picture files were saved. The data from the 6,955,000th row to the 7,945,000th row were also saved in the same manner as in the previous process. The saved files were abnormal graphs, and 11 picture files were saved. In the normal and abnormal graph picture files, the training and test sets were divided at a rate of 60% and 40%. Therefore, 46 randomized graph picture files out of 77 normal graph picture files and 7 randomized graph picture files out of 11 abnormal graph picture files were used as the training set. In other words, 53 normal and abnormal graph pictures were used as the training set, and 35 normal and abnormal graph pictures were used as the test set. The picture files in the training set were RGB image files with a size of (256, 256, 3). Therefore, these images were converted into an array format to enable the learning of the model. By adding a dimension to this array, it was converted to a size of (1, 256, 256, 3), and all image arrays of the training set were stacked to create an array of size (53, 256, 256, 3) as the training set for the CNN model. A label array of size (53, 1) was created by setting the label to zero for a normal graph image and one for an abnormal graph image. The test set underwent the same process as the training set, and a test set array of size (35, 256, 256, 3) and a test label array of size (35, 1) were created. Since the image data have pixel information as a value between zero and 255, normalization was performed by dividing the training set and the test set by 255 to facilitate model learning.

**Figure 6:**
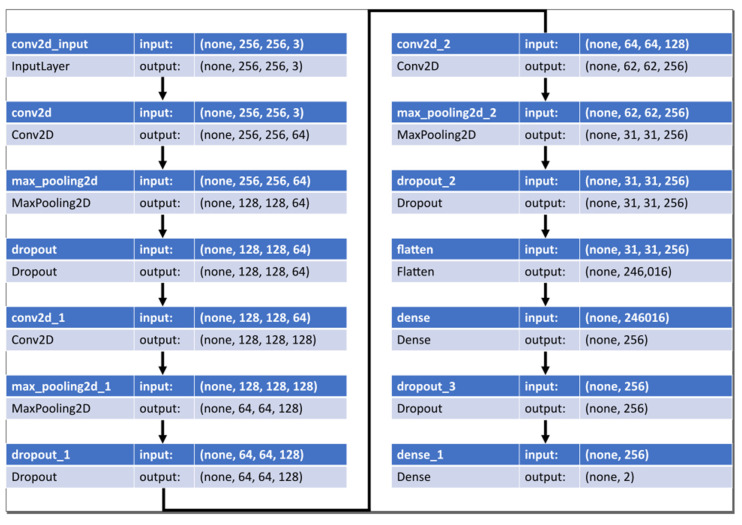
The structure of the CNN model.

The normalized training set goes through the process of Figure 6. The CNN model has three convolutional layers, followed by max-pooling and dropout. Subsequently, the final binary classification was performed using two dense layers. The kernel size of the convolutional layer was set to three, padding was set to the same zero padding, and ReLU was used as the activation function. The max pooling size was set to two, and the dropout probability was set to 0.25.

The outputs of the two neurons in the last layer pass through a sigmoid activation function. Adam was used as the model optimizer, and the accuracy of Equation (9) was used as the performance evaluation metric.

**Figure 7:**
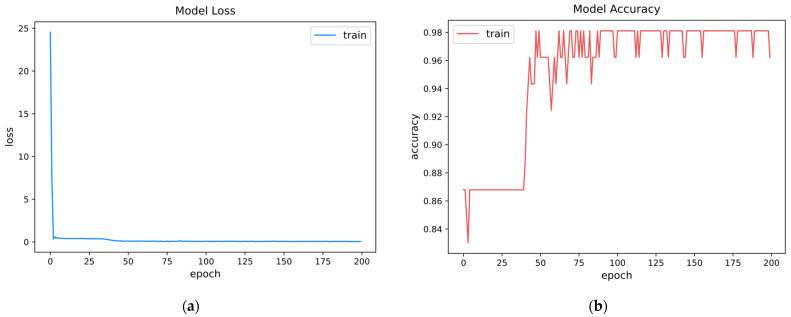
The graphs of the training process and prediction result. (**a**) Training loss; (**b**) model accuracy.

The batch size was set to 30, and the model was trained for 200 epochs. The graphs of the training process and the results are shown in Figure 7. In the model training process, the loss dropped from approximately 24 to 0.3 in 3 epochs and remained below 1 after that; after 42 epochs, the model accuracy increased from approximately 0.87 to more than 0.9 and remained above 0.9 after that. The model accuracy was measured as 0.9714 using the test set.

### 3.6. Anomaly Detection Using CNN Autoencoder

To detect anomalies using the CNN autoencoder, 77 normal pulsating pressure images and 11 abnormal pulsating pressure images generated in Section 3.5 were used. A training set of sizes (77, 256, 256, 3) was created by stacking 77 normal pulsating pressure images of sizes (256, 256, 3). A test set of sizes (11, 256, 256, 3) was created by stacking 11 abnormal pulsating pressure images of sizes (256, 256, 3). The training and test sets were divided by 255 for normalization.

**Figure 8:**
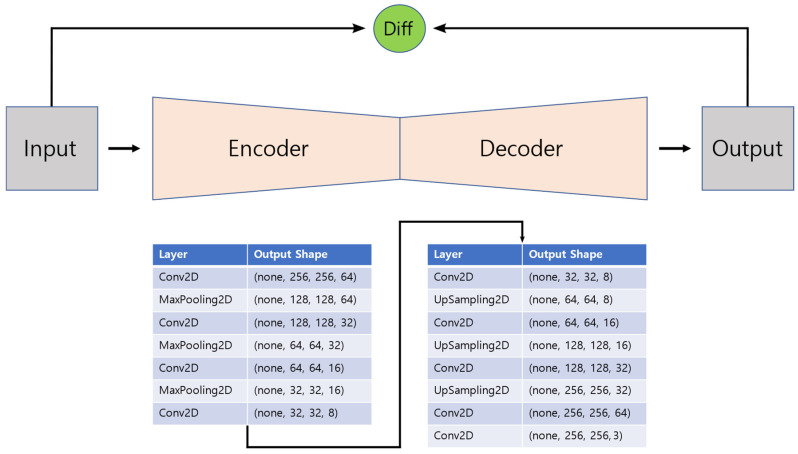
Structure of the CNN autoencoder model for anomaly detection.

The structure of the CNN autoencoder modeled for anomaly detection is shown in Figure 8. An input image of size (256, 256, 3) is input to the model. In the encoder part of the model, convolution was performed while lowering the number of filters to 64, 32, 16, and 8 to reduce the number of feature maps and reduce the horizontal and vertical sizes of the image by max pooling 3 times. In the decoder part of the model, convolution is performed while increasing the number of filters to 8, 16, 32, and 64 to increase the number of feature maps and increase the horizontal and vertical sizes of the image by upsampling 3 times. Finally, convolution was performed to obtain an image of the same size as the input image. The kernel size of the convolutional layer was set to three, and the padding was set to the same zero padding. In the last convolution layer, the sigmoid was used as an activation function, and Swish, as shown in the following Equation (11), was used in the convolution layers:

(11) $$f\left(x\right)=\frac{x}{1+e^{-x}}$$

The max pooling size was set to two, and upsampling was performed using the nearest neighbor method. Nadam was used as the model optimizer, and the training set was used as the input and output data. The number of epochs was set to 100, and the batch size was set to 1 for model training.

**Figure 9:**
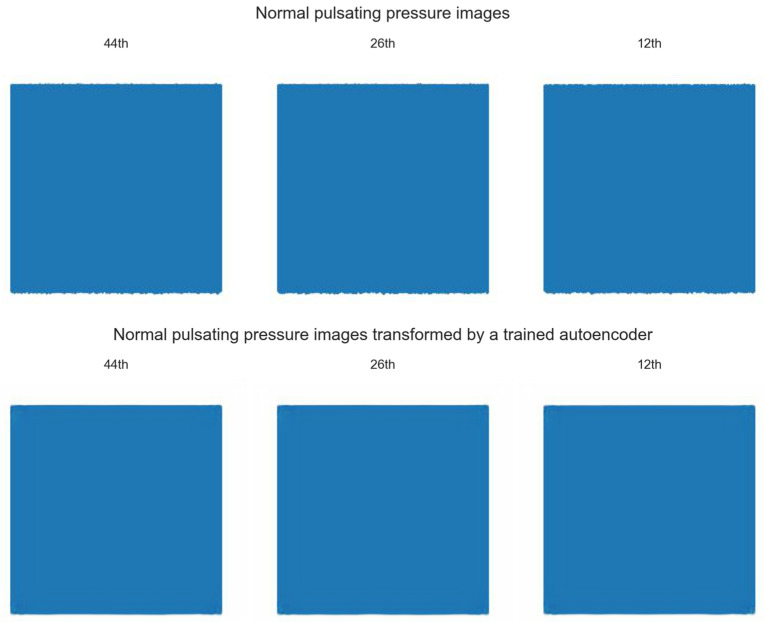
Normal pulsating pressure images and transformed images.

Figure 9 shows 3 randomly sampled images among 77 normal pulsating pressure images and images converted by the CNN autoencoder. As shown in Figure 9, the normal pulsating pressure images transformed by the trained model are almost similar to the existing normal pulsating pressure images. However, there is a slight deviation between the minimum pulsating pressure values in the existing normal pulsating pressure images, and there is almost no deviation in the minimum pulsating pressure values of the normal pulsating pressure images transformed by the CNN autoencoder.

**Figure 10:**
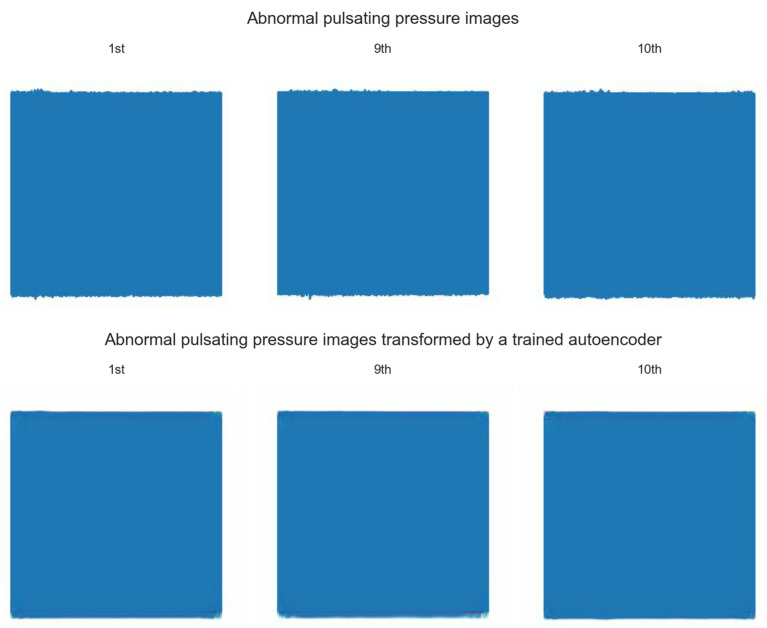
Abnormal pulsating pressure images and transformed images.

Figure 10 shows 3 randomly sampled images among 11 abnormal pulsating pressure images and images converted by the CNN autoencoder. Referring to the images of the abnormal pulsating pressure, it can be seen that a deviation occurs to the maximum and minimum pulsating pressure values. However, in the abnormal pulsating pressure images transformed by the trained CNN autoencoder, the deviation of the pulsating pressure values is removed so that the values of the maximum/minimum pulsating pressures appear as smooth lines. This is because the CNN autoencoder is trained with normal pulsating pressure images with little deviation in pulsating pressure, and even if abnormal pulsating pressure images with a deviation of pulsating pressure are input to the CNN autoencoder, it outputs images similar to normal pulsating pressure images.

**Figure 11:**
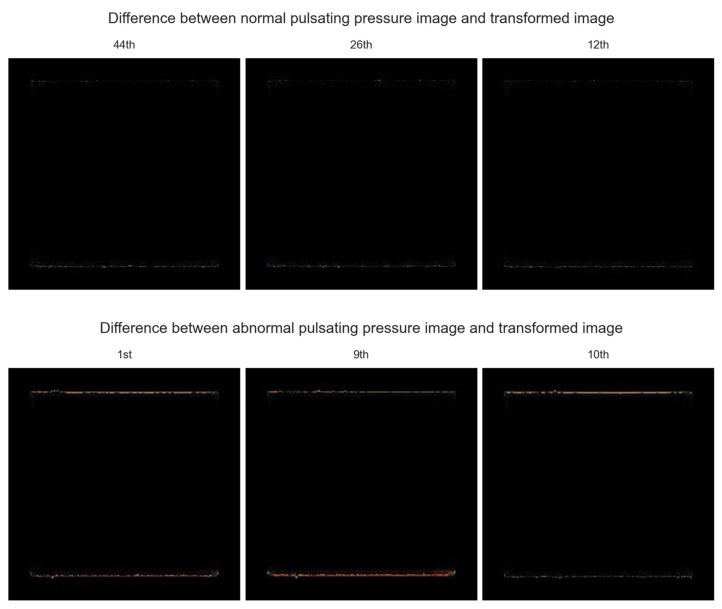
Difference between pulsating pressure images and transformed images.

The upper part of Figure 11 shows the absolute value of the difference between the normal pulsating pressure images in Figure 9 and those transformed by the CNN autoencoder. Since the absolute value of the difference between the normal pulsating pressure image and that transformed by the CNN autoencoder is almost zero, the majority of the images are black. In the maximum/minimum pulsating pressure area, there was no significant difference between the normal pulsating pressure image and that transformed by the CNN autoencoder; hence, it can be observed that there is a slight color in the maximum/minimum pulsating pressure area. Referring to the lower part of Figure 11, the color is conspicuously visible in the maximum/minimum pulsating pressure area, making it possible to detect abnormalities. In Figure 10, due to the difference between the maximum/minimum pulsating pressure area of the abnormal pulsating pressure images and the transformed abnormal pulsating pressure images, it can be observed that a distinct color occurred compared to the upper part of Figure 11.

If the absolute values of the difference between the normal pulsating pressure images and the transformed images are calculated, a NumPy array of sizes (77, 256, 256, 3) is obtained. By averaging the numerical values of 77 images, 77 values were obtained. If the absolute values of the difference between the abnormal pulsating pressure images and the deformed images are calculated, a NumPy array of sizes (11, 256, 256, 3) is obtained. A total of 11 values were obtained by averaging the numerical values of 11 images. The minimum, average, and maximum values of 77 and 11 are listed in Table 4.

**Figure 12:**
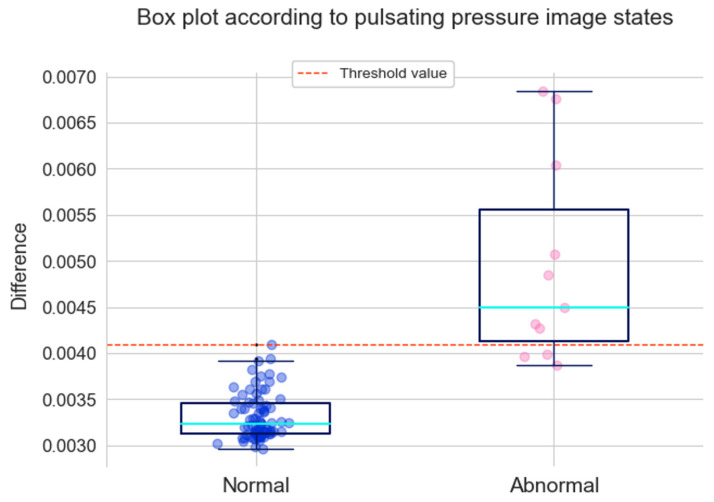
Box plot of CNN autoencoder according to pulsating pressure image states.

The results of the 77 and 11 values displayed as boxplots are shown in Figure 12. The top and bottom lines in the boxplot represent the maximum and minimum values, respectively. The top line in the box is the third quartile, which represents the median of the top 50% of values. The bottom line in the box represents the first quartile, which is the median of the bottom 50% of values. The blue-green line represents the median. The maximum value of the normal condition was used as the threshold for classifying the abnormal conditions. Referring to Figure 12, based on the threshold, it can be confirmed that 8 out of 11 abnormal values were properly detected, except for 3 values.

### 3.7. Anomaly Detection Using LSTM Autoencoder

**Figure 13:**
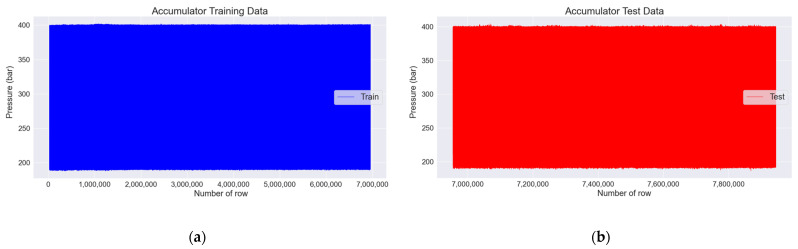
Visualization of pulsating pressure for training and test data. (**a**) Training data consisting of normal pulsating pressure; (**b**) test data consisting of abnormal pulsating pressure.

In Section 3.7, the normal pulsating pressure, i.e., the 25,000th to 6,955,000th row, was used as the training data, as shown in Figure 13a, and the abnormal pulsating pressure, i.e., the 6,955,000th to 7,945,000th row, was used as the test data, as shown in Figure 13b. Accordingly, the training data had 6,930,000 pulsating pressure values, and the test data had 990,000. As shown in Figure 13a, the maximum and minimum values of the pulsating pressure are relatively constant, and when referring to Figure 13b, there is a deviation in the maximum and minimum values of the pulsating pressure. Thus, it can be seen that there are spiking values.

As shown in Equation (12), the min–max scaling was applied to the training and test data. When min–max scaling was performed, the scaled data had a value between zero and one. Equation (12) is as follows:

(12) $$x_{scaled}=\frac{x-x_{min}}{x_{max}-x_{min}}$$

To be input into the LSTM cell, the training data were converted to size (6930000, 1, 1), and the test data were converted to size (990000, 1, 1).

**Figure 14:**
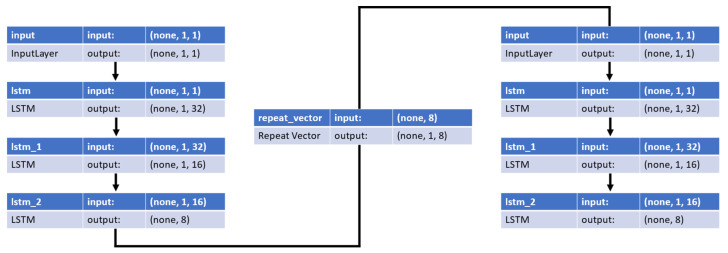
Structure of the LSTM autoencoder model.

The structure of the LSTM autoencoder used to detect anomalies is shown in Figure 14. The left part of Figure 14 shows an encoder that generates compressed input data. In the encoder, the LSTM cells were sequentially composed of 32, 16, and 8 cells, and Swish was used as the activation function. The middle part of Figure 14 shows a repeat vector layer that distributes the compressed representational vector across the time steps of the decoder. The right side of Figure 14 shows the decoder that provides the reconstructed input data. In the decoder, 8, 16, and 32 LSTM cells were used, and Swish was used as the activation function. The LSTM autoencoder model uses Nadam as an optimizer and the MAE shown in Equation (13) as a loss function. Here, MAE is a performance metric that converts the difference between actual and predicted values into absolute values and averages them. Equation (13) is as follows:

(13) $$MAE=\frac{1}{n}\;\overset{n}{\underset{i=1}{\sum }}\left|x_{i}-x\right|$$

**Figure 15:**
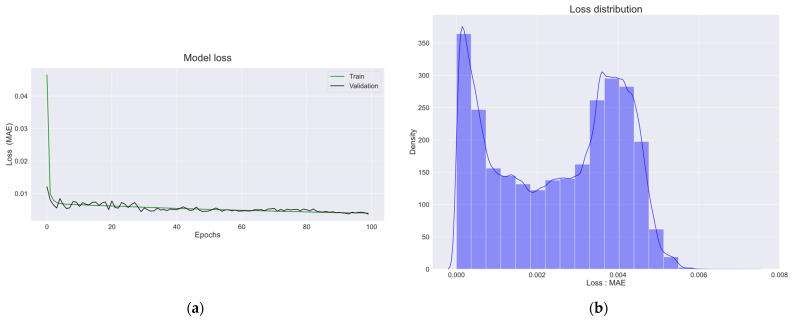
(**a**) Loss graphs for the model’s training and validation sets; (**b**) loss distribution graph of the trained LSTM autoencoder model.

The model was trained for 100 epochs with a batch size of 1000 and a validation set of 5% of the training set. Figure 15a shows the training and validation sets trained for 100 epochs. As shown in Figure 15a, the MAE values for the training and validation sets decreased as the number of epochs increased, indicating that the model was well-trained.

Figure 15b shows the distribution of MAE values calculated using the actual and predicted values of the training set of the trained LSTM autoencoder model. Referring to Figure 15b, the MAE values were mostly zero. The density of MAE decreased from 0 to 0.002 of the MAE value, and it increased again from 0.002 to 0.004. It can be seen that the density of MAE starts to decrease from 0.004 of the MAE value, and the density becomes almost zero at 0.006.

**Figure 16:**
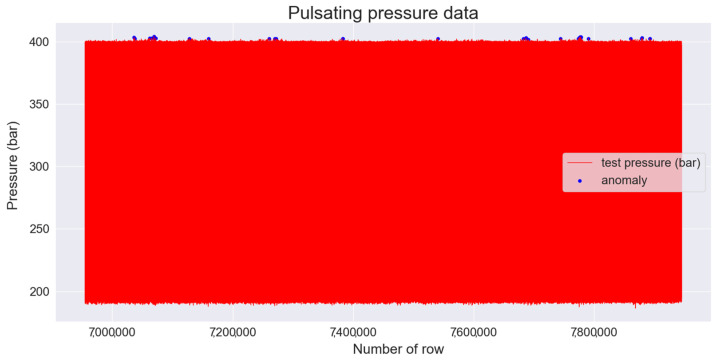
Anomaly detection graph based on the threshold of the LSTM autoencoder in the test set.

Anomalies were detected using the largest MAE value obtained by the training set as the threshold, and the obtained threshold value was 0.0073. To detect anomalies in the test set, the MAE value was obtained from the values predicted by the test set and the trained LSTM autoencoder. When the obtained MAE value exceeded the threshold, an abnormality was detected, and 36 data points were detected. The 36 data points that detected anomalies in the test set are indicated by blue dots in Figure 16.

### 3.8. Comparison of Six Anomaly Detection Algorithms

In Section 3.8, the six algorithms mentioned above were compared and analyzed. Threshold averaging algorithms detected anomalies based on maximum and minimum thresholds. However, as shown in Figure 5, it could detect anomalies well at the maximum threshold, but not well at the minimum threshold. Therefore, depending on the data acquired, the selection of a maximum or minimum threshold may be necessary. Unlike threshold averaging algorithms, the five algorithms mentioned later are models trained by training data.

Here, SVM and XGBoost used the same training set and test set and, when comparing accuracy, XGBoost was more robust. The CNN model required a process of saving images, but the accuracy was 0.9714, which was significantly higher than both SVM and XGBoost. The CNN autoencoder detected anomalies using the images used in the CNN model and correctly classified 8 out of 11 abnormal images as abnormal images. This model has the advantage of being able to visualize the anomalous detected area through the difference between the image that passed through the autoencoder and the image that did not. In the case of the LSTM autoencoder model, time series data was used, and 36 data points exceeding the threshold were detected among the entire test set.

## 4. Conclusions

Hydraulic accumulators are important devices used in hydraulic systems in various industries. However, in diaphragm-type hydraulic accumulators, the problem of damage caused by continuous abnormal pulsating pressure was raised, and anomaly detection algorithms were developed to prevent this in advance. Based on the abnormal pulsating pressure, six anomaly detection algorithms were developed, and the results are as follows.

The first developed algorithm detects anomalies based on the average of the maximum and minimum threshold values. It was found that the anomaly detection was predicted 2.5 h before the hydraulic accumulator was broken. The SVM and XGBoost models detect anomalies using training and test sets divided based on the time point at which the maximum/minimum threshold deviation occurs in the pulsating pressure graph. Here, SVM and XGBoost had accuracies of 0.8571 and 0.8857, respectively, on the test set.

The fourth algorithm detects anomalies using the CNN technique. The training and test sets used in the SVM and XGBoost models were saved as images and applied to the CNN model. The accuracy of the trained CNN model for the test set is 0.9714. The fifth developed algorithm is a CNN autoencoder model, and images of the training and test sets used in the CNN model were used. The threshold was calculated based on the difference between the predicted and input images of the CNN autoencoder model for the training set, and it was found that 8 out of 11 images were detected as abnormal for the test set. The sixth developed algorithm is the LSTM autoencoder. The training and test sets used in the LSTM autoencoder model had the same reference point as the SVM and XGBoost models for dividing the training and test sets. The LSTM autoencoder also detected anomalies by obtaining a threshold based on the difference between the predicted and input values, and it was confirmed that 36 data points were abnormally detected in the test set.

This study confirmed that an accident could be prevented in advance before breakage by installing a digital pressure sensor in a marine hydraulic accumulator and detecting an abnormality using various anomaly detection models.

## Figures and Tables

**Table 1:** Specification of the digital pressure sensor.

Parameters	Values
Maker	Sensys
Measuring range	−0.1–500 MPa
Accuracy	±0.5–0.8% FS
Compensated temperature range	−10–70 °C
Operating temperature range	−20–80 °C
Proof pressure	≤150 MPa: X1.5 or 150 MPa, whichever is less. >150 MPa: X1.5 or 500 MPa, whichever is less.
Burst pressure	≤150 MPa: X2 or 150 MPa, whichever is less. >150 MPa: X2 or 600 MPa, whichever is less.
Vibration	49.1 m/s^2^ {5G}, 10–500 Hz
Shock	490 m/s^2^ {50G}

**Table 2:** Confusion Matrix.

		**Predicted Values**	
		Predicted Positive	Predicted Negative
Actual Values	Actual Positive	True Positive (TP)	False Negative (FN)
	Actual Negative	False Positive (FP)	True Negative (TN)

**Table 3:** Hyperparameters used in the XGBClassifier model and their values.

Parameters	Values
eta (learning rate)	0.3
min_child_weight	1
Gamma	0
max_depth	6
colsample_bytree	1
Lambda	1
Alpha	0
scale_pos_weight	1

**Table 4:** Minimum, average, and maximum values of the differences between pulsating pressure images and transformed images.

Condition	Parameters	Values
Normal	Minimum	0.0030
	Average	0.0033
	Maximum	0.0041
Abnormal	Minimum	0.0039
	Average	0.0050
	Maximum	0.0068

## Data Availability

Not applicable.

## Nomenclature

CNN	Convolutional neural network
LSTM	Long short-term memory
MAE	Mean absolute error
Nadam	Nesterov-accelerated adaptive moment estimation
RBF	Radial basis function
ReLU	Rectified linear unit
SVM	Support vector machine
XGBoost	Extreme gradient boosting
